# Mutations and Evolution of the SARS-CoV-2 Spike Protein

**DOI:** 10.3390/v14030640

**Published:** 2022-03-19

**Authors:** Nicholas Magazine, Tianyi Zhang, Yingying Wu, Michael C. McGee, Gianluca Veggiani, Weishan Huang

**Affiliations:** 1 Department of Pathobiological Sciences, School of Veterinary Medicine, Louisiana State University, Baton Rouge, LA 70802, USA; nmagaz1@lsu.edu (N.M.); tzhang10@lsu.edu (T.Z.); mmcgee9@lsu.edu (M.C.M.); 2 Center of Mathematical Sciences and Applications, Harvard University, Cambridge, MA 02138, USA; ywu@cmsa.fas.harvard.edu; 3 The Donnelly Center for Cellular and Biomolecular Research, University of Toronto, Toronto, ON M5S 3E1, Canada; gianluca.veggiani@utoronto.ca; 4 Department of Microbiology and Immunology, College of Veterinary Medicine, Cornell University, Ithaca, NY 14853, USA

**Keywords:** SARS-CoV-2, COVID-19, spike, mutation, evolution, infectivity, immune escape

## Abstract

The SARS-CoV-2 spike protein mediates target recognition, cellular entry, and ultimately the viral infection that leads to various levels of COVID-19 severities. Positive evolutionary selection of mutations within the spike protein has led to the genesis of new SARS-CoV-2 variants with greatly enhanced overall fitness. Given the trend of variants with increased fitness arising from spike protein alterations, it is critical that the scientific community understand the mechanisms by which these mutations alter viral functions. As of March 2022, five SARS-CoV-2 strains were labeled “variants of concern” by the World Health Organization: the Alpha, Beta, Gamma, Delta, and Omicron variants. This review summarizes the potential mechanisms by which the common mutations on the spike protein that occur within these strains enhance the overall fitness of their respective variants. In addressing these mutations within the context of the SARS-CoV-2 spike protein structure, spike/receptor binding interface, spike/antibody binding, and virus neutralization, we summarize the general paradigms that can be used to estimate the effects of future mutations along SARS-CoV-2 evolution.

## 1. Introduction

Of the structural proteins that comprise the SARS-CoV-2 virus, it is likely that none is as well-studied as the spike (S) protein. The S protein is critical for the function of SARS-CoV-2, being the protein responsible for target recognition, cellular entry, and endosomal escape [1]. Given the multifaceted nature of the S protein, it is not surprising that the enhanced fitness seen in many variants of SARS-CoV-2 has been attributed to mutations of the S protein [2].

Perhaps one of the best exemplars of the impacts of mutational changes in the SARS-CoV-2 S protein is the D614G mutation. Mutation D614G was first identified in mid-2020 and rapidly spread throughout the global population, with over 95% of all sequenced SARS-CoV-2 variants containing this mutation by January 2021. Today, the D614G mutation is found within all major circulating strains of SARS-CoV-2 and has been attributed to substantially increasing the infectivity of the virus [3].

The newly identified SARS-CoV-2 variant of concern (VOC) as of December 2021, Omicron, has a total of 34 mutations (30 nonsynonymous mutations, 3 deletions, and 1 insertion) relative to the wild-type S protein, accounting for 2.5% of all amino acids occurring within the protein [4]. Given the rapid rate with which these mutations are appearing, it is now more important than ever for the scientific community to understand the potential mechanisms by which these alterations are positively selected along SARS-CoV-2 evolution. This review addresses the understood mechanisms by which these mutations likely enhance the infectivity and/or the immunity-escaping ability of the virus, while consolidating general paradigms that can be utilized to estimate the mechanisms by which other mutations will likely arise during SARS-CoV-2 evolution.

## 2. Structure of the SARS-CoV-2 Spike Protein

To comprehend the mechanisms by which mutations of the S protein are able to enhance infection and/or immune escape, it is critical to understand the general structure and function of S as a whole. The SARS-CoV-2 S protein comprises two subunits, S1 and S2, which can be subdivided into two and five primary subdomains, respectively [5]. The S protein as a whole is responsible for target recognition, binding, and cellular entry by SARS-CoV-2, with S1 and S2 playing distinct roles during this process [6]. The S1 subunit is responsible for target recognition and binding, while S2 is involved in membrane fusion and endosomal escape.

**Figure 1:**
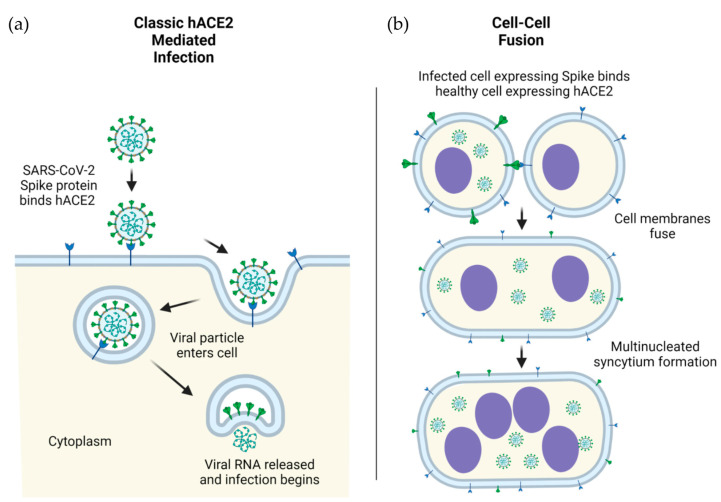
ACE2-mediated cellular infection by SARS-CoV-2. (**a**) Schematic of direct cellular entry of SARS-CoV-2 viral particles into human cells, mediated by ACE2. (**b**) Cellular infection by ACE2-spike mediated cell-cell fusion. Infection in human (h) cells is used as an example.

The S1 subunit contains an N-terminal domain (NTD) and a C-terminal receptor-binding domain (RBD). The RBD (~21 kDa) is responsible for the recognition of the angiotensin-converting enzyme 2 (ACE2) which acts as the receptor for SARS-CoV-2 viral entry [7]. The RBD recognizes a number of other structurally related targets, though the RBD’s role in recognition of these receptors is not yet well-understood in the context of disease progression, symptoms, and severity [8]. In contrast to the RBD, the NTD of S1 is underinvestigated and therefore less well-characterized. The NTD plays a critical role in overall S protein structural conformation, and mutations occurring in the NTD are linked to SARS-CoV-2 immune escape [9]. The NTDs of related coronaviruses are capable of facilitating infection via the recognition of sugar-containing molecules such as glycoproteins, although the exact role of this potential binding is debated in the context of SARS-CoV-2 [10]. The primary mechanism of SARS-CoV-2 initial infection is viral entry mediated by S (on virus) and ACE2 (on host cells) interactions in humans (Figure 1a) as well as in model organisms such as nonhuman primates.

The S2 subunit contains a fusion peptide (FP) subdomain, two heptad repeat subdomains (HR1 and HR2), a transmembrane subdomain, and a C-terminal tail [10]. After initial ACE2 recognition and viral attachment, the FP subdomain wedges into the cellular membrane, at which point the HR1 and HR2 subdomains are pulled toward one another in an antiparallel confirmation [11]. After contact, the HR1 and HR2 subdomains form a six-helical bundle, causing the viral particle as a whole to come into close proximity to the cell, ultimately leading to membrane fusion and viral entry [12]. The transmembrane subdomain anchors the S to the envelope of SARS-CoV-2, while the C-terminal tail sits inside the viral particle. Though the role of the transmembrane subdomain is primarily structural, the C-terminal tail was demonstrated to promote S escape from the endoplasmic reticulum [13]. This escape of S from the endoplasmic reticulum likely leads to the aggregation of S on the surface of infected cells, which can interact with the ACE2 receptor expressed on neighboring cells, ultimately leading to cell–cell fusion, syncytia formation, and spread of the viruses from infected cells to neighboring cells [14] (Figure 1b).

## 3. SARS-CoV-2 Variants of Concern

**Figure 2:**
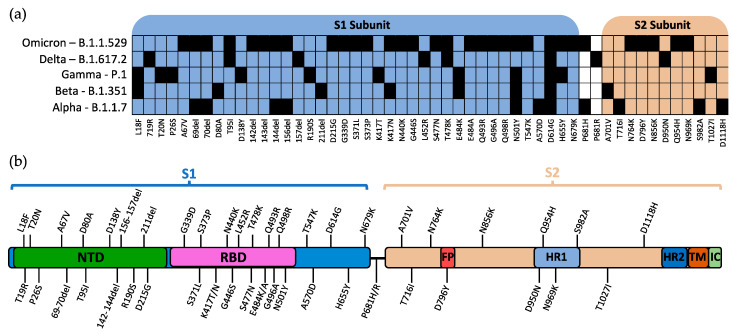
Mutations of spike protein within VOCs. (**a**) Defining mutations of WHO-labelled VOCs and their relative position in the S1/S2 subunits. (**b**) Visual representation of the relative position of mutations within the SARS-CoV-2 spike protein. Mutations are reported as listed in the CoV-Lineages database as of December 2021. NTD: N-terminal domain; RBD: receptor-binding domain; FP: fusion peptide; HR1: heptad repeat 1; HR2: heptad repeat 2; TM: transmembrane region; IC: intracellular domain.

The World Health Organization (WHO) currently designates five strains of SARS-CoV-2 as variants of concern (VOC): Alpha, Beta, Gamma, Delta, and Omicron [15]. The Pango lineages for these variants are B.1.1.7, B.1.351, P.1, B.1.617.2, and B.1.1.529, respectively [16]. The defining mutations for each SARS-CoV-2 variant and their relative location within S are shown in Figure 2a,b, respectively.

SARS-CoV-2 strains are given a VOC designation on the basis of either a demonstrated increase in transmissibility or an increase/alteration in disease presentation [15]. Although the pathogenicity and overall mortality of these strains are debatable, it is generally accepted that all five VOCs have enhanced transmissibility (with increased infectivity and/or immune escape) compared to the earlier “wild-type” SARS-CoV-2 identified in the Wuhan outbreak during early 2020 [17,18,19]. In particular, the most recent dominant variant, Omicron, displays advantageous fitness in transmission and immune escape, suggestive of potential serotype demarcation [20]. The overall increased fitness for positive selection of these variants is further supported by the fact that 6 of the 52 total mutations that can be found among these variants existed in over half of all sequenced genomes as of January 2022.

## 4. Mutations of the S1 Subunit Containing the Receptor-Binding Domain

**Figure 3:**
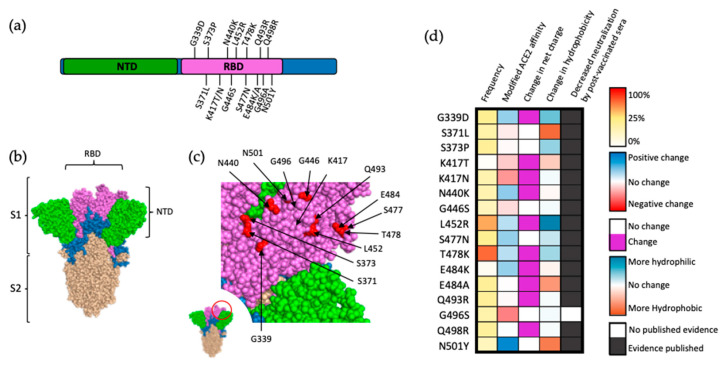
Mutations within the RBD of SARS-CoV-2 spike protein. (**a**) Visual representation of mutations within the RBD (magenta) of SARS-CoV-2 spike. Notably, 15 out of the 32 amino acid substitutions in the spike protein are localized in the RBD. (**b**) Structural location of SARS-CoV-2 S subunits. (**c**) Close-up view of RBD (magenta) and VOC-occurring mutations (red). (**d**) Frequency, effect on ACE2 affinity, modification of charge at physiological pH, alterations in hydrophobicity at pH 7, and direct evidence of decreased neutralization by postvaccinated sera for mutations within the RBD of S protein. Frequency is presented as a percentage of reported SARS-CoV-2 genomes logged within the GISAID database, as a notion of fitness advantage. Alteration in ACE2 affinity based on data by Starr et al. [21], with mutations that increase ACE2 affinity in blue and mutations negatively impacting ACE2 affinity in red. Frequency represented as a percentage of reported SARS-CoV-2 genomes logged within the GISAID database retrieved 10 March 2022. Alterations in hydrophobicity based on previously established values [23]. Alterations in residue charge based on standard calculations at physiological pH.

Due to the role of the RBD in ACE2 recognition and binding, it stands to reason that changes in the amino acid sequence of RBD can dramatically impact S binding affinity for ACE2 and, ultimately, SARS-CoV-2 infectivity. This is reflected by the fact that while mutations occur throughout this region, most of them are located on the surface of S (Figure 3a), allowing for direct interactions with potential ligands (Figure 3b,c). A 2020 deep mutational analysis conducted by Starr et al. screened for alterations of human ACE2 affinity that occurred as a result of single-site mutations within this region [21]. Comparing the results of this study to variants of concern that have arisen as of March 2022 (Figure 3a), among sixteen mutations that contributed to reduce the neutralization of the mutant viruses by post-vaccinated sera, only seven likely conferred increased binding affinity of S for ACE2 (G339D, N440K, L452R, S477N, T478K, E484K, and N501Y). These data suggest that binding affinity between S and the host receptor is not the predominant factor contributing to the positive selection of mutations within the RBD [21]. Polymorphism of ACE2 is rare (mean Fst 0.0167), with alterations hypothesized as modifying RBD-ACE2 affinity being exceedingly uncommon, even within this subset [22].

An alternative explanation for positive selection of mutations within the RBD of SARS-CoV-2 is enhancement of resistance to postvaccinated sera [24]. Studies testing monoclonal antibodies isolated from SARS-CoV-2-vaccinated individuals have demonstrated resistance conferred by nearly all RBD mutations [19,25,26,27,28] (Figure 3d). This is not surprising as 40% of the antibodies produced against SARS-CoV-2 target the RBD, and the vast majority of these antibodies are neutralizing antibodies [29]. Additionally, the majority of mutations within this region alter either the charge or hydrophobicity of the RBD, dramatically increasing the probability of antibody escape via modified epitope affinity or local conformational changes decreasing epitope accessibility. An interesting exception to this trend of individual mutations showing increasing resistance to postvaccinated sera is represented by the G496A substitution, though this may be due to this mutation being fairly recent and data on its effects being limited at this time. Taken together, it can be inferred that the primary driver of positive selection arising from the majority of mutations within the RBD is enhanced neutralization resistance as opposed to increased affinity of S to ACE2.

Thirteen of the sixteen RBD mutations associated with VOCs are found within the Omicron variant, ten of which exclusively occur in the defining sequence of Omicron. A popular theory for the sudden appearance of these mutations (many of which reduce ACE2 affinity) is that the Omicron strain evolved in an immunocompromised patient, thereby reducing selective pressure and allowing for multiple concurrent mutations as the strain developed [30]. This theory is further supported by the strain first being identified in an immunocompromised patient in South Africa [30]. An alternate theory for the sudden appearance of this multitude of mutations is the occurrence of zoonosis. Five of the defining Omicron mutations (K417, E484, Q493, Q498, and N501) are commonly found in mouse-adapted SARS-CoV-2 strains, suggesting that the strain may have initially developed in animal species (for example, murine) and then was transmitted to and further evolved within the human population [31]. These observations demonstrate the possibility that not all individual COVID-19 mutations arise as a result of normal selective pressures, but may occur as the culmination of net-positive concurrent mutations that arise under atypical conditions.

## 5. Mutations of the S1 Subunit N-Terminal Domain

**Figure 4:**
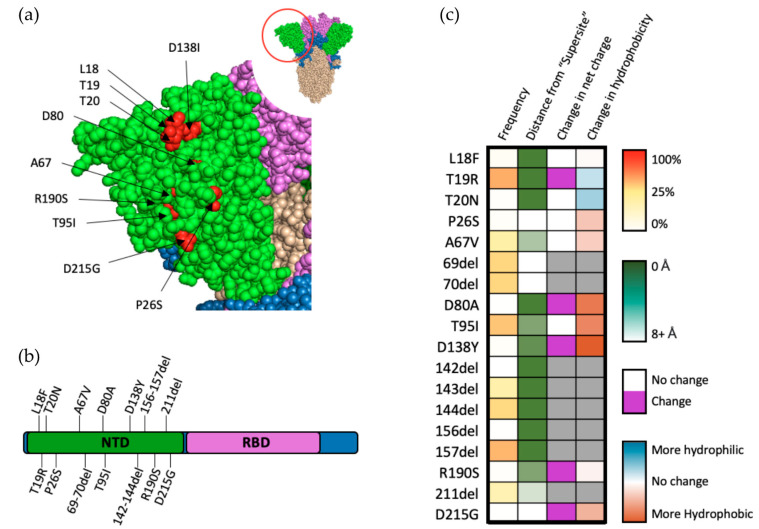
Mutations within the NTD of SARS-CoV-2 spike protein. (**a**) Structural representation of substitutions (red) within the NTD (green) of SARS-CoV-2 spike protein. (**b**) Visual representation of mutations within the NTD of spike protein. (**c**) Frequency, residue distance from NTD “supersite”, modification in charge at physiological pH, and change in hydrophobicity at pH 7. Residue distance was calculated in PyMOL on the protein three-dimension structure (PDB-6ZGG), by measuring the distance between the nearest atom of “supersite” amino acids identified by Mccalum et al., 2021 [32] and the nearest atom of amino acids of interest.

The reasons for the positive selection of variants bearing mutations within the NTD of SARS-CoV-2 S are multifaceted, with common mutations occurring throughout the NTD subdomain (Figure 4a,b). Though 35% of antibodies targeting SARS-CoV-2 target the NTD, only about one-third of these antibodies have a neutralizing effect [29]. Further investigation of neutralizing antibodies targeting NTD have revealed a “supersite” to which nearly all of these antibodies bind [32]. An interesting hallmark of antibodies targeting the NTD region is their ability to decrease cell–cell fusion, suggesting that the NTD may play a role in syncytium formation [32]. Fourteen of the eighteen mutations that occur in the NTD of VOCs occur within close proximity (eights angstroms) of this antigenic supersite (Figure 4c). Taken together with the fact that many of these mutations occur within the Omicron variant (which appeared only after vaccinations became widely distributed), it is possible that resistance to neutralizing antibodies (particularly those found in postvaccinated sera) targeting the NTD play a large role in the positive selection for SARS-CoV-2 [33].

Though they do not occur near the NTD neutralization “supersite”, T95I and Δ69–70 occur in almost one-quarter of all sequenced genomes and are indicators of the highly successful Omicron variant, which has demonstrated a clear fitness advantage, suggesting positive selection for these mutations. Analysis of Δ69–70 in pseudoviral models revealed a substantial ability for this mutation set to increase infectivity [34]. Further analysis demonstrated that this increase is primarily mediated by enhancement of cell–cell fusion, while simultaneously having little to no effect on neutralization by NTD-neutralizing antibodies [34]. Similarly, analysis of metadata for qPCR cycling thresholds obtained from patients infected with SARS-CoV-2 showed an increase in viral load for patients with variants bearing the T95I mutation, especially in the presence of Δ142 [35]. Structural modelling additionally revealed topological changes that may occur in the NTD “supersite” as a result of T95I, suggesting that it is possible for other mutant residues, even if they are not in the supersite region, to alter the topology of the supersite and affect SARS-CoV-2 neutralization by postvaccinated sera [35].

## 6. Other S1 Mutations and Mutations Occurring between S1 and S2

Mutations that occur outside of major subdomains such as the NTD and RBD also heavily influence SARS-CoV-2 infectivity and/or sensitivity to sera from convalescent and/or vaccinated individuals. Although they do not occur in major subdomains, all residues within this region substantially alter local charge and/or hydrophobicity, qualities that greatly increase the likelihood of modifying local protein structure and, ultimately, function. This observation suggests that these mutations have arisen as a result of positive selection as opposed to random genetic drift.

**Figure 5:**
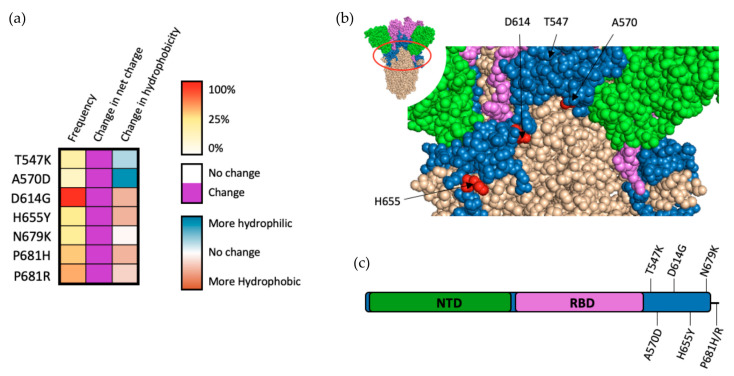
Mutations occurring outside of major SARS-CoV-2 spike subdomains. (**a**) Frequency, charge modifications, and alteration in hydrophobicity for mutations occurring outside of major spike subdomains. (**b**) Structural representation of mutations occurring within this region (P681 is not shown, as it is on the other surface of the current 3D view). (**c**) Schematic representation of mutations occurring outside of major spike subdomains.

D614G is the most ubiquitous of all known SARS-CoV-2 mutations of the S protein, having occurred in over 99% of all sequenced cases of COVID-19 as of 2022 (Figure 5a). While D614G does not occur firmly within any particular subdomain (Figure 5b,c), it seems to have an effect on multiple aspects of the S protein. Initial pseudoviral models for infection demonstrated that D614G greatly enhances SARS-CoV-2 infectivity, likely resulting from increased incorporation of S into the SARS-CoV-2 virion [36]. Further analysis of D614G demonstrated that the mutation alters the conformation of the RBD, increasing its occurrence in an “up” state that enhances the binding affinity between S and ACE2, as well as increasing S accessibility by furin [37].

As the pandemic has progressed, mutations at P681 have emerged in the majority of sequenced SARS-CoV-2 genomes. These mutations are particularly interesting as they occur in a 10-residue stretch from amino acids 680–689, which comprise the furin cleavage site which develops during viral particle production. Structural modeling of P681 mutations have demonstrated that alterations at this site are capable of increasing furin cleavage [38]. In pseudoviral models, only P681R has been demonstrated to independently increase cellular infectivity via furin cleavage, while P681H does not appear to significantly impact either furin cleavage or viral infectivity independently [39,40]. The A570D mutation may also impact furin cleavage, with structural analysis of this alteration showing an increase in the spacing between individual chains of S trimer (brought about by drastic changes in both charge and hydrophobicity), potentially enhancing furin accessibility [41]. Pseudoviral testing of the A570D was unable to demonstrate that the mutation can independently increase infectivity, with the mutation destabilizing the SARS-CoV-2 pseudovirion [42]. This finding indicates that positive selection of A570D may occur as a result of interaction with coinciding mutations that are known to stabilize SARS-CoV-2 pseudovirions (such as D950N and D1118H) [42].

## 7. Mutations of the S2 Subunit

**Figure 6:**
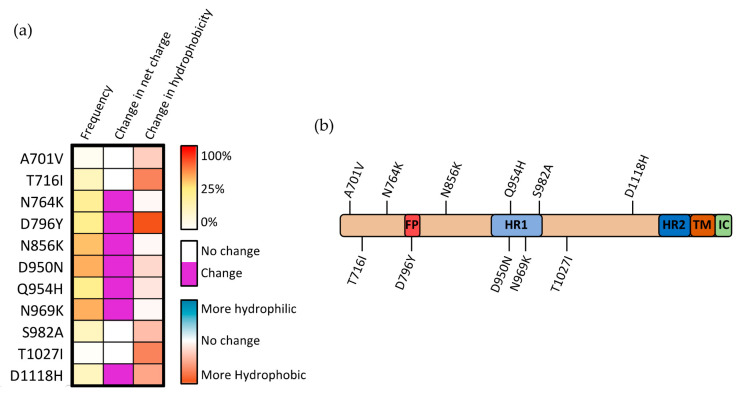
Mutations occurring within the S2 subunit of SARS-CoV-2 spike protein. (**a**) Frequency, modification in charge at physiological pH, and alteration in hydrophobicity for mutations occurring within the S2 subunit. (**b**) Visual representation of mutations occurring within the S2 subunit.

The mechanisms through which mutations within the S2 subunit affect SARS-CoV-2 infectivity are potentially diverse, with the S2 subunit containing five subdomains, each having a distinct function. Only four mutations within the S2 region occur commonly in SARS-CoV-2 VOCs other than the recent Omicron strain (and are therefore better researched): T716I, D950N, S982A, and D1118H (Figure 6a). Two of these three mutations (D950N and S982A) lie within the HR1 domain, suggesting that alterations within this region may be particularly prone to driving positive selection (Figure 6b). This positive selection may occur by altering the association of HR1 with HR2, the possibility of which is further enhanced when considering that both mutations substantially alter either the charge or hydrophobicity at these sites. Structural analysis of the region between the heptad repeat domains (where D1118H occurs) showed that residues within this region play an important role in repositioning the S2 domain postfusion, allowing for the heptad repeat domains to interact with the targeted cell membrane [43].

The S982A substitution increases the presentation of the “up” RBD state by eliminating the interaction with T547, which stabilizes the “down” RBD state. This change in RBD state is partially counteracted by the complementary A570D mutation that occurs in the Alpha SARS-CoV-2 variant [44]. It was demonstrated that the D570 residue is capable of forming an interprotomer hydrogen bond with N856, effectively re-establishing the bond that stabilizes this “down” confirmation [44]. Within the S trimer, when D1118H mutation occurs, the three histidine residues (one from each monomeric S) form a histidine triad within the trimer, stabilizing the overall structure of the trimeric S complex [45]. Although there is, as yet, no direct evidence of the role this stabilization plays, it was suggested that this effect may compensate for local destabilizations caused by associated mutations such as T716I [44]. The concurrence of these conflicting mutations within the same variants indicates that there is likely a balancing act between maintaining S stability and allowing for the switching between various conformations in the pre- and postfusion states.

## 8. Conclusions

Mutations within the S protein of the circulating variants of SARS-CoV-2 are increasing at a significant rate and are likely to occur more often as selective pressures from host immunity gained in previous infections and/or vaccinations continue to drive rapid evolution. Although many mutations have presented over the course of the pandemic, diligent research has elucidated a general trend: many of the emerging and surviving mutants enhance SARS-CoV-2 functions regarding infectivity and immune escape. With new variants constantly emerging, future therapies, as well as vaccinations, will be more successful if they demonstrate effectiveness over a broad range of S variants. Given these rapid changes, it may also be beneficial to give particular consideration to therapies that function independently of S structure and functionality.

## Data Availability

Statistics pertaining to SARS-CoV-2 variant and sequence frequencies were taken from the GISAID repository https://www.gisaid.org/ (accessed on 10 March 2022). The furin cleaved spike protein of SARS-CoV-2 with one RBD erect structure (PDB, 6ZGG) [46] was used to draw the 3D structure of spike in this review article. PyMOL (The PyMOL Molecular Graphics System, Version 2.0, Schrödinger, LLC, New York, NY, USA) was used to render the spike 3D structure in all figures and measure the residue distances in Figure 4.
