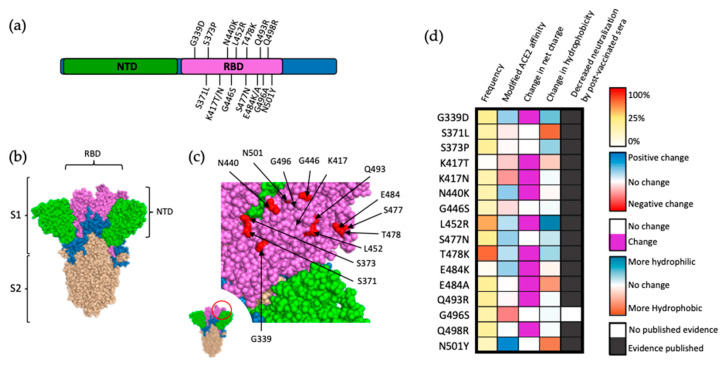# Correction: Magazine et al. Mutations and Evolution of the SARS-CoV-2 Spike Protein. *Viruses* 2022, *14,* 640

**DOI:** 10.3390/v15091787

**Published:** 2023-08-23

**Authors:** Nicholas Magazine, Tianyi Zhang, Yingying Wu, Michael C. McGee, Gianluca Veggiani, Weishan Huang

**Affiliations:** 1 Department of Pathobiological Sciences, School of Veterinary Medicine, Louisiana State University, Baton Rouge, LA 70802, USA; nmagaz1@lsu.edu (N.M.); tzhang10@lsu.edu (T.Z.); mmcgee9@lsu.edu (M.C.M.); 2 Center of Mathematical Sciences and Applications, Harvard University, Cambridge, MA 02138, USA; ywu@cmsa.fas.harvard.edu; 3 The Donnelly Center for Cellular and Biomolecular Research, University of Toronto, Toronto, ON M5S 3E1, Canada; gianluca.veggiani@utoronto.ca; 4 Department of Microbiology and Immunology, College of Veterinary Medicine, Cornell University, Ithaca, NY 14853, USA

## 1. Error in Figure

In the original publication [1], there was a mistake in Figure 3c as published. The right edge of Figure 3c was accidentally cropped out. The corrected Figure 3c appears below. The authors state that the scientific conclusions are unaffected. This correction was approved by the Academic Editor. The original publication has also been updated.